# High-fat-diet induced development of increased fasting glucose levels and impaired response to intraperitoneal glucose challenge in the collaborative cross mouse genetic reference population

**DOI:** 10.1186/s12863-015-0321-x

**Published:** 2016-01-05

**Authors:** Hanifa J. Abu-Toamih Atamni, Richard Mott, Morris Soller, Fuad A. Iraqi

**Affiliations:** Department of Clinical Microbiology and Immunology, Sackler Faculty of Medicine, Tel-Aviv University, Ramat Aviv, Tel-Aviv, 69978 Israel; University of Oxford, Oxford, UK; Hebrew University, Jerusalem, Israel

**Keywords:** Type 2 diabetes (T2D), Metabolic syndrome, Obesity, High fat diet (HFD), Sex effects, Collaborative Cross mouse reference population, Heritability, Coefficient of genetic variation

## Abstract

**Background:**

The prevalence of Type 2 Diabetes (T2D) mellitus in the past decades, has reached epidemic proportions. Several lines of evidence support the role of genetic variation in the pathogenesis of T2D and insulin resistance. Elucidating these factors could contribute to developing new medical treatments and tools to identify those most at risk. The aim of this study was to characterize the phenotypic response of the Collaborative Cross (CC) mouse genetic resource population to high-fat diet (HFD) induced T2D-like disease to evluate its suitability for this purpose.

**Results:**

We studied 683 mice of 21 different lines of the CC population. Of these, 265 mice (149 males and 116 females) were challenged by HFD (42 % fat); and 384 mice (239 males and145 females) of 17 of the 21 lines were reared as control group on standard Chow diet (18 % fat). Briefly, 8 week old mice were maintained on HFD until 20 weeks of age, and subsequently assessed by intraperitoneal glucose tolerance test (IPGTT). Biweekly body weight (BW), body length (BL), waist circumstance (WC), and body mass index (BMI) were measured. On statistical analysis, trait measurements taken at 20 weeks of age showed significant sex by diet interaction across the different lines and traits. Consequently, males and females were analyzed, separately. Differences among lines were analyzed by ANOVA and shown to be significant (*P* <0.05), for BW, WC, BMI, fasting blood glucose, and IPGTT-AUC. We use these data to infer broad sense heritability adjusted for number of mice tested in each line; coefficient of genetic variation; genetic correlations between the same trait in the two sexes, and phenotypic correlations between different traits in the same sex.

**Conclusions:**

These results are consistent with the hypothesis that host susceptibility to HFD-induced T2D is a complex trait and controlled by multiple genetic factors and sex, and that the CC population can be a powerful tool for genetic dissection of this trait.

**Electronic supplementary material:**

The online version of this article (doi:10.1186/s12863-015-0321-x) contains supplementary material, which is available to authorized users.

## Background

In the past decades, the prevalence of Type 2 Diabetes Mellitus (T2D) has reached epidemic proportions. Epidemiologic surveys show that to date, about 171 million individuals have T2D worldwide, with projections of 366 million by 2030 [1, 2]. The increased incidence of T2D coincides with an increase in obesity and metabolic syndrome (MTS). With a lag of about a decade, we are now seeing the beginning of a far worse obesity/MTS/T2D epidemic in Asia and South America similar to that observed as Native American peoples adopted Westernized lifestyles [3]. T2D, obesity and MTS are a heterogeneous group of metabolic disorders characterized by defects of both insulin secretion and insulin activity.

Several lines of evidence provide support for the role of genetic variation in the pathogenesis of T2D and insulin resistance [4–7]. Indeed, large resources dedicated to investigate genetic epidemiology of T2D using data of thousands of patients and matched control populations, have identified numerous quantitative trait loci (QTL) that affect susceptibility to T2D and MTS [8, 9]. Identification of the underlying genes may help clarify the relationship of T2D and MTS [10, 11].

In addition to environmental and genetic predisposition risk factors, sex-related differences were recognized in the pathogenesis of T2D [12]. Men and women experience T2D and MTS, differently. In spite of multiplied expenses and time demands when conducting research with both sexes, it is crucial to consider the basic sex differences for providing new sex-specific approaches in prevention, diagnosis, and treatment of T2D and related MTS features [13, 14].

The laboratory mouse is one of the most important tools at our scientific disposal in understanding mammalian gene function. The scientific community has taken advantage of the fundamental similarity of mouse and man; at the genetic level (99 % of mouse genes turn out to have analogues in humans [15–17]), and mice and men share similar physiology and anatomy. In genetically defined strains of mice, chromosomal regions responsible for the genetic variation of complex traits can be mapped as QTL in experimental populations available for precise study under defined conditions [18–20]. Once QTL have been identified, genetic analysis can be extended successfully to humans [21, 22]. It has been suggested that the variation among extant strains of mice can be used for mapping QTL associated with susceptibility to chronic and infectious diseases [23–27]. In this context, T2D can be considered as a complex trait disease, where the challenge is the high fat diet. Several previous studies have shown that mouse strains differ substantially in their metabolic phenotype under normal relatively low-fat diet (LFD) conditions and in response to a high-fat diet (HFD) and QTLs affecting these traits have been mapped based on these mouse strains [28–33]. In a previous linkage analyses study, QTLs associated with dietary obesity were identified, by using the C57BL/6byj X 129P3/J F2 mouse model [34]. They found that allelic effects differed greatly depending on sex and diet, demonstrating the importance of sex in the determination of dietary obesity in the mouse. Nevertheless, the use of existing mouse strains to identify QTLs affecting T2D is limited by the close relationships among the existing moue strains, which limits the spectrum of QTL that can be uncovered, and does not permit the high-resolution mapping needed for positional cloning of the underlying genes. The Collaborative Cross (CC) genetic reference population (GRP), now in advanced stages of development by a community effort of the Complex Trait Consortium (CTC) was designed to remove these limitations (CTC, www.complextrait.org). The CC was created by full reciprocal 8-way matings of 8 divergent strains of mice: A/J, C57BL/6 J, 129S1/SvImJ, NOD/LtJ, NZO/HiLtJ, CAST/Ei, PWK/PhJ, and WSB/EiJ. The founder strains of this population include three wild-derived inbred mouse strains (CAST/Ej, PWK/hJ and WSB/EiJ), which introduce enormous genetic and phenotypic diversity [35–37], while the designed structure of the CC lines allows the QTLs underlying this diversity to be mapped with high power and high resolution [38–41].

A cohort of CC RIL is now under development in our laboratory at Tel Aviv University. As a first step towards mapping genetic factors affecting HFD-induced T2D development in CC mice, we have assessed 21 CC lines after 12 weeks on a HFD (42 % fat content) and subsequently measured their Glucose tolerance by means of fasting blood glucose and intraperitoneal glucose tolerance test (IPGTT). In parallel, a control cohort of 17 of the same 21 CC lines, were maintained on standard Chow diet (18 % fat). In contrast to most previous studies, in our study we have equally assessed both sexes of the CC lines.

It is well documented that there is a strong relationship between overall Obesity (Body weight and BMI) and Central Obesity (Waist circumference), and Type 2 Diabetes development. These reports show that overall obesity by body weight (BW), body length (for BMI calculation) and central obesity by waist circumference (WC) can be strong predictors for T2D development [43–45]. In the current study, we measured overall Obesity by Body weight in grams and Body Mass Index (BMI), while Central Obesity was measured by Waist Circumference (WC). Type 2 Diabetes is a silent disease that progress over long time at pre-symptomatic phase, yet early detection of preclinical disease is possible through monitoring of the glycemic stage of the body. Higher levels of Fasting Glucose and GTT are strongly associated with prediabetic conditions [46, 47]. Glycemic stages range from Normoglycaemia stage (low risk) to diabetic stage, while prediabetic stage (high risk) is located in between. Glycemic stage in our study was measured via fasting blood glucose (6 h fasting) and via calculation of Area under curve across 180 min of a Glucose tolerance test (IPGTT),

In the present study, we show that the HFD induces increased body weight, waist circumference, BMI and fasting glucose levels, and impairs Glucose tolerance. We use these data to infer broad sense heritability of the traits adjusted for number of mice tested in each line; coefficient of genetic variation; genetic correlations between the same trait in the two sexes, and phenotypic correlations between different traits in the same sex.

## Methods

All experimental mice and protocols were approved by the Institutional Animal Care and Use Committee of Tel-Aviv University (approval numbers: M-07-084 and M-012-025).

### Collaborative cross mouse population

A total of 683 mice from 21 CC lines were used in the study. In this experiment, we used large number mice, so there will enough mice and power per line in each diet and sex, as well to reduce the differences within a line and obtain accurate traits. Of these, 265 mice (149 males and 116 females; average, 7.09 males and 5.52 females per line) were tested for HFD challenge, and 384 mice (239 males and 145 females; average 11.95 males and 8.55 females per line) from 17 of the 21 lines were used as control cohort on standard Chow diet. The difference in numbers between sexes and diets is a matter of the mice that were available at the time. The mice were provided by the Small Animal Facility at Sackler Faculty of Medicine at Tel-Aviv University. The CC lines were at inbreeding generations F_10_-F_25_, with a minimum of 90 % homozygosity as determined by extensive high-density genotyping. Full details of the development of these CC lines are given in Iraqi et al. [39].

### Determining the number of mice used in the study

This study had three objectives: (1) To show that a HFD could cause development of biomarkers for T2D and MTS in the CC mouse reference population with emphasis on uncovering gender x diet interaction effects; (2) To demonstrate sufficient genetic variation in development of T2D and MTS biomarkers among different lines of the CC population to justify using the CC lines for mapping of the relevant QTL; and (3) To (eventually) characterize individual CC lines with respect to the multi-trait development of these biomarkers For Objective (2) it is clear that 10 lines would not be sufficient to obtain convincing estimates of genetic variation among the CC lines, while 50 lines is more than needed. This leads to choice of 20 lines. For Objective (3) it is clear that 1 or 2 animals per line x gender combination would be too few to characterize individual combinations for multi-trait comparisons, while 15–20 would probably be too much. We took 8–10 as our goal. This led to planned totals of about 320–400 animals per treatment. Thus, for objective (1), we had well over 100 animals for each diet x gender combination. This should be sufficient to detect effects of magnitude 0.25 s.d.u. with α = 0.05 and β = 0.20.

### High fat dietary challenge

Mice were maintained from weaning (3 weeks of age) until 8 weeks of age on the standard rodent Chow diet TD.2018SC (3.1 kcal/gm), which consists of 18 % Kcal from fat, 24 % from protein, and 58 % from carbohydrates (Teklad Global, Harlan Inc., Madison, WI, USA). From 8 to 20 weeks, a cohort of mice was challenged by a high-fat Western diet TD 88137 (4.5 kcal/gm), which consists of 42.0 % Kcal from fat, 15.3 % from protein, and 42.7 % from carbohydrates (primarily sucrose) (Teklad Global, Harlan Inc., Madison, WI, USA). During this challenge period, the control cohort continued to be maintained on Chow diet. Mice had free access to water and diet during the entire period.

### Phenotyping

Body weight (BW), body length (BL), and body waist circumference (WC) were measured bi-weekly for each animal in the HFD cohort. At the end of 12 weeks dietary challenge, BW, BL and WC and intraperitoneal glucose tolerance test were assessed for all animals from both diets,

### Intraperitoneal glucose tolerance test (IPGTT) and fasting glucose

Mice were fasted for 6 h (6:00 AM −12:00 AM) with free access to water. After 6 h fasting, blood glucose levels were measured at time zero, before a solution of glucose (2.5 g glucose per kg mouse) was administered by intraperitoneal (IP) injection [42, 48, 49]. Afterwards, the blood glucose level was monitored by tail bleeding at time 0, 15, 30, 60, 120 and 180 min after glucose injection, using U-RIGHT glucometer TD-4267 (TaiDoc Technology Corporation 3 F, 5 F, No.127, Wungong 2nd Rd., 24888 Wugu Township, Taipei County, Taiwan).

### Area under curve for glucose tolerance (AUC)

An area under the curve (AUC) trapezoid model from 0 to 180 min after challenge was used to quantitatively evaluate glucose clearance activity. AUC between any two time points was calculated as (Time difference in minutes between sequential reads)*(Glucose level 1st time point + Glucose level 2nd time point)/2). In all cases, glucose level is measured from the level at time zero to the end time point level (180 min).

### Body weight (BW), Waist circumference (WC), Body length (BL), and body mass index (BMI)

BW was measured with accuracy within 0.1 g. WC in cm (accuracy to 0.1 cm) was taken manually around the belly area midway between hip and thorax. BL in cm (accuracy 0.1 cm) was taken manually nose to base of the tail. BMI was calculated as BMI = BW/BL^2^

### BW, WC, and BMI gain

BW, WC, and BMI gains were calculated as the difference between the challenge end time-point value (20 weeks old) minus the initial value (8 weeks old). Individual data points were obtained at 8 weeks only for mice on HFD, while at 20 weeks for all mice from both diets. Since CC lines are highly inbred, the phenotypic values are more or less interchangeable. On this assumption the mice on Chow diet were assigned initial 8-week values randomly from the pool of tested mice. This enabled calculations for gain to be made for these animals as well.

### Data analysis

Statistical Analysis was performed using the statistical software package SPSS Version 22 (IBM SPSS Statistics 22).

***Two-way ANOVA by sex and diet*** examined the independent effects of Sex and Diet and their interaction on the different measured phenotypes. Data from all of the lines for each Sex x Diet combination were pooled.

***One-way ANOVA by lines*** was carried out separately for each of the four Sex x Diet combinations. This provided data on significance of the differences among lines, and for estimating broad sense heritability (H^2^) and coefficient of genetic variation (CVg).

***Pearson Correlation*** coefficients between the different measured traits were calculated by SPSS.

### Broad sense heritability and the genetic coefficient of variation (“Evolvability” parameter)

The phenotypes measured in the present study all fall into the category of “Quantitative” (or “Complex”) traits. Such traits typically display considerable phenotypic variation (Vp) among the individuals of a population. When analyzed appropriately this variation can be decomposed into two sources, a genetic component of variation (Vg) and an environmental component (Ve). Thus, Vp = Vg + Ve. In principle, the genetic component includes direct (“additive”) effects of the genes, and effects of dominance, epistasis, and gene x environment interactions. Heritability refers to the proportion of phenotypic variation among individuals that is contributed by the genetic component of variation.

$$ \mathrm{Heritability} = \mathrm{V}\mathrm{g}/\mathrm{V}\mathrm{p} $$

Estimates of Vg from many types of experimental populations and analyses include only additive genetic effects. In this case, the heritability estimate is termed a “narrow-sense” heritability, denoted h^2^. If Vg includes anything more than additive effects, the heritability estimate is termed a “broad-sense” heritability, denoted H^2^. In the CC populations, Vg includes epistatic and gene x environmental effects, and hence it is a “broad sense” heritability. In the present study, H^2^ was calculated from the results of the One-Way ANOVA, as

$$ {H}^2 = {V}_g/\left({V}_g + {V}_e\right), $$

where,

*H*^*2*^ is the broad sense heritability for a particular diet x sex combination*,*

*V*_*g*_ is the genetic variance component estimated from the ANOVA for that combination as (MS_between_ – *V*_*e*_)/n

*V*_*e*_ is the environmental variance component, estimated from the above ANOVA as MS_within_

n is the average number of mice per line for the particular diet x sex combination.

For example, consider the population composed of the combined 149 male mice of the 21 CC lines on HFD diet (i.e., the HFD x male-sex combination). The heritability estimate for end-BW from the One-way ANOVA of this population (0.47, Table 1), measures the proportion of total phenotypic variation among these 149 mice that is contributed by genetic factors segregating among the 21 lines. Full details of estimating trait heritability in our CC lines were presented elsewhere [50].

**Table 1:** Broad sense heritability (H^2^) and genetic coefficient of variation (CVg) of the tested traits under high fat diet (HFD, 42 % fat) and chow (CH, 18 % fat) diets, separately for females and males. Initial, end, trait value at start 8 weeks) and end (20 weeks) of HFD challenge period. Gain, end trait-value minus initial trait-value. Total AUC, area under the curve for intraperitoneal glucose tolerance test between 0 and 180 min from start of test. ND, not done (see text for explanations). Table 1 also shows H^2^n calculated for the average animals per line (n) values of our data: 5.52 for females-HFD, 7.09 for males-HFD, 8.55 for females-Chow, and 11.95 for males-Chow ^a^

Trait	Females						Males					
	CH Diet			HF Diet			CH Diet			HF Diet		
	H^2^	H^2^n	CVg	H^2^	H^2^n	CVg	H^2^	H^2^n	CVg	H^2^	H^2^n	CVg
BW Initial	0.37	0.89	0.08	0.37	0.76	0.08	0.37	0.92	0.09	0.37	0.81	0.09
BMI Initial	0.47	0.92	0.14	0.47	0.83	0.14	0.33	0.90	0.09	0.33	0.78	0.09
WC Initial	0.54	0.94	0.13	0.54	0.87	0.13	0.38	0.92	0.10	0.38	0.81	0.10
BW End	0.57	0.95	0.14	0.56	0.88	0.16	0.55	0.96	0.16	0.47	0.86	0.15
BMI End	0.40	0.90	0.12	0.57	0.88	0.16	0.52	0.95	0.13	0.43	0.84	0.16
WC End	0.46	0.92	0.12	0.48	0.84	0.16	0.25	0.86	0.07	0.29	0.74	0.13
BW Gain	0.53	0.94	ND	0.51	0.85	ND	0.33	0.90	ND	0.38	0.81	ND
BMI Gain	0.56	0.94	ND	0.32	0.72	ND	0.24	0.85	ND	0.36	0.80	ND
WC Gain	0.56	0.94	ND	0.46	0.82	ND	0.11	0.70	ND	0.17	0.59	ND
Fasting Glucose	0.38	0.89	0.13	0.26	0.66	0.16	0.44	0.94	0.17	0.24	0.69	0.13
Total AUC	0.42	0.91	0.15	0.43	0.81	0.27	0.24	0.85	0.17	0.37	0.81	0.21
Mean	0.49	0.93	0.13	0.47	0.83	0.16	0.33	0.90	0.12	0.36	0.80	0.13

^a^ No. of animals (in parentheses, mean number of animals per line): HFD, males and females, 21 lines, 116 females (5.52), 149 males (7.09); Chow diet, numbers varied somewhat according to sex and trait. On average there were for males 20 lines average 11.95 mice per line; for females 17 lines, average 8,55 mice per line

We used the Genetic Coefficient of Variation (also termed, the “evolvability” parameter) the ratio of the genetic standard deviation (V_G_^0.5^) to the mean across all CC lines) as a unit-free measure of genetic variation for comparison among traits [51, 52].

$$ C{V}_G = {V_G}^{0.5}/ Mean, $$

where,

*V*_*G*_ is as defined above, and

Mean, is the unweighted mean trait value for the particular diet x sex combination across all CC lines.

CV_G_ is of interest as providing a benchmark for judging whether *V*_*G*_ values of traits in the CC lines are large or small relative to *V*_*G*_ values normally found in segregating populations [53].

### Heritability of line means (H^2^n)

The heritability of the trait in the study population tells us the correlation between the observed phenotype of an individual in that population and the true genetic value of the individual. Since the heritability is usually less than 1.0, this means that an individual with given observed phenotype, can have a true genetic value that varies more or less widely about that phenotype. The statistical parameter that determines the potential variation in genetic value, of an individual chosen on the basis of its phenotypic value, is the “coefficient of Non-determination” (also termed the “coefficient of Alienation”) equal to (1-H^2^). For a trait with H^2^ = 0.5, this means that the genetic value of an individual can vary about its observed phenotypic value with a variance equal to half of the genetic variance of the entire population.

Often we are interested in identifying individual lines showing high or low expression of a trait of interest, for follow up physiological or genetic studies. In this case, we choose the line of interest on the basis of the observed mean trait value of the line. In this case, we denote by H^2^n the proportion of variation among the line means that is due to genetic factors,. H^2^n is generally considerably greater than H^2^, according to the following expression, based on Robertson [54]:

$$ {\mathrm{H}}^2\left(\mathrm{n}\right) = {\mathrm{nH}}^2/\left(1 + \left(\mathrm{n}\hbox{-} 1\right){\mathrm{H}}^2\right) $$

Where, n is the mean number of individuals tested per line. Examination of the expression shows that H^2^n becomes large rapidly with increase in n. That is, adding more mice within each line gives us better and better mean estimates of line genetic value until 100 % of the variation in line means is explained by genetics (i.e. H^2^n → 1 as n → ∞). For example, end-BW of male mice on HFD had H^2^ = 0.47 on an individual mouse basis, but there were on average 7.09 male mice per line, giving a value of H^2^n = 0.86, meaning that 86 % of the variation in line means results from genetic factors. The coefficient of non-determination for line means is only 0.16 in this case, so that true line means vary in a narrow band about the observed line mean.

### Genetic and phenotypic correlations

The basic expression defining phenotypic and genetic correlations among traits is derived in Falconer and Mackay [55]. Using our notation for heritability and a * to indicate multiplication, the expression has the following form,

$$ \mathrm{rPxy} = \mathrm{H}\mathrm{x}*\mathrm{H}\mathrm{y}*\mathrm{rGxy} + \mathrm{Ex}*\mathrm{E}\mathrm{y}*\mathrm{rExy}, $$

where,

rPxy is the phenotypic correlation between the two traits X and Y based on individual measurements (not on line means) of the two traits in the same individuals.

rGxy and rExy are genetic and environmental correlations between the two traits respectively,

Hx and Hy are square root of heritabilities (H^2^x and H^2^y) for X and Y, respectively,

Ex and Ey are square root of coefficients of non-determination (1-H^2^x) and (1-H^2^y), respectively,

Examination of the expression shows that if H^2^x and H^2^y are high, rPxy will be primarily determined by rGxy, while if H^2^x and H^2^y are low, rPxy will be primarily determined by rExy.

When correlations are based on means of lines, the expression takes the form

$$ \mathrm{rPnxy} = \mathrm{H}\mathrm{x}\mathrm{n}*\mathrm{H}\mathrm{y}\mathrm{n}*\mathrm{rGxy} + \mathrm{E}\mathrm{x}\mathrm{n}*\mathrm{E}\mathrm{y}\mathrm{n}*\mathrm{rExy}, $$

where,

rPnxy is the phenotypic correlation between trait X and trait Y based on means of n individual per line.

rGxy is the genetic correlation, as before,

Hxn and Hyn are square root of heritabilities (H^2^xn) and (H^2^yn) of means of lines for the traits X and Y, where H^2^xn and H^2^yn are calculated as in the previous section.

Exn and Eyn are square root of coefficients of non-deermination (1-H^2^xn) and (1-H^2^yn) for traits X and Y respectively.

Recall that H^2^n becomes larger with increasing n. It necessarily ensues that E^2^n and its square root become progressively smaller. Consequently, as n increases, the expression Exn*Eyn*rExy becomes negligibly small, and rPnxy ~ Hxn*Hyn*rGxy.

### Genetic correlation between the same trait in the two sexes

The genetic correlation between the same trait in the two sexes tells us the extent to which the same genetic factors are operating in males and females. This is a special case of the above, in which the same trait is measured in different individuals (males and females, respectively). Consequently, there will not be any environmental correlation between the two measurements, since they are taken on independent individuals with independent history of life events affecting the traits. Thus, when correlations are based on means of lines for the same trait in the two sexes (male, m; female, f), the expression takes the form

$$ \mathrm{rPnmf} = \mathrm{Hxnm}*\mathrm{Hxnf}*\mathrm{rGmf} $$

where

rPnmf is phenotypic correlation between the trait X in males and trait X in females based on means of n individual per line x sex combination.

rGmf is the genetic correlation between Trait X in males and Trait X in females.

Solving for rGmf, we obtain,

$$ \mathrm{rGmf} = \mathrm{rPnmf}/\left(\mathrm{Hxnm}*\mathrm{Hxnf}\right) $$

Hxnm and Hxnf are square root of heritabilities (H^2^xnm) and (H^2^xnf) of means of lines for the trait X, where H^2^xnm and H^2^xnf are calculated as in the previous section, separately for males (H^2^xnm) and females (H^2^xnf). For example, from Table 2 we find phenotypic correlation under HFD between line means for end BW for males and line means for end BW for females = 0.734. From Table 1 we have H^2^nf for end BW = 0.88 and H^2^nm = 0.86. Taking square roots, we have rGmf = 0.734/(0.938*0.927) = 0.845.

**Table 2:** Phenotypic (rPnmf) and genetic (rGmf) correlations between the same traits in males and females on high fat diet (HF Diet)**,** based on line means for the given trait in males and females ^a^. BW, Body Weight; BMI, Body Mass Index; WC, Waist circumference; Total AUC, total area under curve of the intraperitoneal glucose tolerance test; Initial, measurements at experiment start-point age of 8 weeks; End, end time- point of the experiment after 12 weeks high-fat dietary challenge; Gain, difference between end and initial time-point values

HF Diet		
Trait	rPnmf	rGmf
BW Initial	0.626	0.797
BMI Initial	0.148	0.184
WC Initial	0.607	0.723
BW End	0.734	0.845
BMI End	0.705	0.819
WC End	0.414	0.525
BW Gain	0.646	0.776
BMI Gain	0.163	0.215
WC Gain	0.532	0.761
Fasting Glucose	0.338	0.500
Total AUC	0.542	0.672

^a^ No. of animals: 21 lines all HFD, 149 males, 116 females

## Results

### Two-way ANOVA, least square estimated effects by diet and sex

Table 3 shows least squares estimated mean values for the tested traits by diet and sex and their interaction, as analzyed by two-Way ANOVA with diet and sex as main effects. These are global effects of sex and diet and their interaction, based on the combined data across all lines. As will be seen, there are major differences among the different lines in these effects*.* Because of very strong sex x diet interaction effects, the estimates of the main effects of diet and sex provided by the Two-way ANOVA analysis, are not meaningful. Hence, these main effect estimates are not presented or discussed further.

**Table 3:** Least square estimated mean values for tested traits by diet and sex and their interaction. Dietary challenge from age 8–20 weeks. C, Chow diet (18 % fat); H, High fat diet (42 % fat); M, male; F, female; CM, males on chow diet; CF, females on Chow diet; HM, males on high-fat diet; HF, females on high-fat diet. Above, estimated mean; in parentheses below, standard error (SE). Values in the same row that share the same superscript letter do not differ significantly at *P* < 0.05. GxD, significance of Sex x Diet interaction. RIF, increase on HFD relative to Chow, females; RIM, increase on HFD relative to Chow, males. NS, not significant. ND, not done, as data are prior to HFD treatment ^e^. BW, Body Weight; BMI, Body Mass Index; WC, Waist circumference; Total AUC, total area under curve of the intraperitoneal glucose tolerance test; Initial, at experiment start-point age of 8 weeks; End, end time-point of the experiment after 12 weeks high-fat dietary challenge; Gain, difference between end and initial time-point values

Trait	CF	HF	CM	HM	SXD	RIF	RIM	RIM/RIF
BW Initial	18.87^a^	18.92^a^	22.99^b^	22.92^b^				
	(0.31)	(0.27)	(0.24)	(0.24)	NS	ND	ND	ND
BMI Initial	0.218^a^	0.216^a^	0.244^b^	0.243^b^	NS	ND	ND	ND
	(0.005)	(0.004)	(0.004)	(0.003)				
WC Initial	5.90^a^	5.86^a^	6.69^b^	6.64^b^		ND	ND	ND
	(0.11)	(0.1)	(0.09)	(0.09)	NS			
BW End	23.44^a^	25.52^b^	28.70^c^	34.35^d^				
	(0.65)	(0.55)	(0.5)	(0.49)	***	1.089	1.197	1.099
BMI End	0.230^a^	0.230^a^	0.260^b^	0.310^c^				
	(0.006)	(0.005)	(0.005)	(0.004)	***	1	1.192	1.192
WC End	6.77^a^	6.58^a^	7.92^b^	8.74^c^				
	(0.17)	(0.15)	(0.13)	(0.13)	***	0.927	1.103	1.19
BW Gain	4.27^a^	6.53^c^	5.70^bc^	11.42^d^				
	(0.56)	(0.48)	(0.43)	(0.42)	***	1.529	2.004	1.31
BMI Gain	0.011^a^	0.014^a^	0.020^a^	0.063^b^				
	(0.007)	(0.006)	(0.005)	(0.005)	***	1.273	3.15	2.474
WC Gain	0.87^ab^	0.72^a^	1.23^b^	2.09^c^				
	(0.18)	(0.15)	(0.14)	(0.14)	***	0.827	1.699	2.055
Fasting Glucose	146.15^a^	176.26^c^	163.17^bc^	206.58^d^				
	(3.86)	(6.65)	(3.35)	(5.62)	NS	1.206	1.266	1.05
Total AUC	25701.2^a^	37497^c^	32923.1^b^	53004.9^d^				
	(1126.7)	(1224.4)	(1069.7)	(1080.4)	***	1.459	1.61	1.103

^e^ No, of animals: for BW, BMI, WC: Chow Diet; females 16 lines, 85 animals; males, 20 lines 141 animals; HFD, females 21 lines 116 animals, males 21 lines 149 animals. For Fasting Glucose and AUC: Chow diet, 17 lines 137 females, 146 males; HFD 21 lines, 116 females, 149 males

***, *P* < 0.001

#### Initial trait values

Since all animals were raised on Chow until 8 weeks of age, there were no differences between the animals assigned to the Chow diet treatment and those assigned to the HFD for initial BW, WC or BMI. There were, of course significant differences at this age between males and females assigned to the different diet groups. As can be expected, females had lower BW and BMI and smaller WC.

#### End trait values

For females, end-BW differed significantly on HFD compared to Chow, but the differences were not large. End-WC and end-BMI did not differ significantly and actual differences were very small. For all three traits, end values in males were significantly greater under HFD than under Chow diets. This striking difference between behavior of males and females, was expressed as a highly significant sex x diet interaction term in the ANOVA.

#### Trait gain values

For females, BW gain under HFD was significantly greater (+52.9 %) than under Chow diet. WC and BMI did not differ significantly under the two diet treatments. For all three traits, male gains were markedly greater under HFD than under Chow diet (BW, +100.3 %; BMI, +215.0 %; WC, +69.9 %). Here too, the stronger response of males to HFD resulted in a highly significant interaction effect.

#### Fasting glucose values

Fasting blood glucose levels were higher on Chow diet for males than for females, and increased strongly on HFD both for males and for females. Thus, in this instance a significant interaction effect was not prsent.

*AUC values* show very much the same pattern as BW gain. Females on HFD showed values significantly greater than on Chow (+45.8 %), but their response was exceeded by the males on HFD (+61.0 % relative to males on Chow). Thus, here again the powerful interaction of diet and sex manifested, with males showing a much stronger response to HFD than females.

### Relative change on HFD compared to Chow for males and females

Examination of the values in Table 3 shows that in many cases, the absolute increase in trait value under HFD compared to Chow seems to stand in proportion to the trait value under Chow. That is, when trait values under Chow are low, the increase from Chow diet to HFD is low, and when Chow levels are high, the increase is high. Since male trait values under Chow are generally greater than female trait values, this alone can generate an interaction effect between sex and diet. To explore this further, Table 3 also shows the relative increase in HFD compared to Chow for females (RIF) and for males (RIM) and their ratio (RIM/RIF). If increase on HFD is proportional to Chow levels, then RIM/RIF will equal 1.0, indicating that males and females are responding in the same proportional manner when Chow levels are taken into account. In this event, the source of the interaction effect is the difference in starting Chow values for males and females. Examination of Table 3, shows that this is indeed the case for fasting glucose levels, and to some extent for end BW and for AUC. But for all other traits, in particular BMI gain and WC gain, the effect of HFD in males is greater than in females, even when standardized against starting Chow values, indicating a true sex x diet interaction. Thus, under HFD challenge, at the physical level, females and males respond very differently: female mice remain lean, while males become obese, But at a deeper biochemical level, both sexes appear to react the same, showing more or less equivalent proportional increases in fasting glucose and IPGTT AUC levels.

### Variation among lines for end-BW, fasting blood glucose and IPGTT AUC

**Fig. 1:**
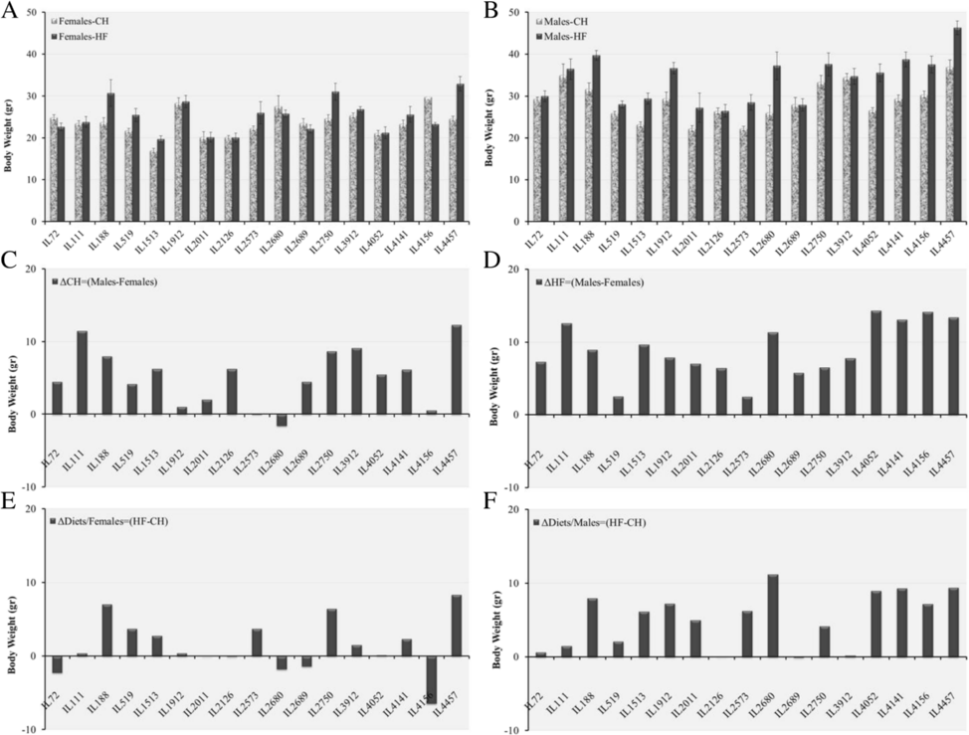
End time point Body Weight (g) of 17 CC lines separately for females and males, after 12 weeks on Chow diet (CH, 18 % fat) and on high fat diet (HFD, 42 % fat). Bar graphs **a** and **b** show end time point Body Weight means (±SE) for CH and HFD of females and males, respectively, by lines. Bar graphs **c** and **d**, show diference between males and females for Chow (ΔCH = Males-Females) and HFD (ΔHF = Males-Females), respectively. Bar graphs **e** and **f** show differences between CH and HFD for females (ΔDiets/Females = HF-CH) and males (ΔDiets/Males = HF-CH), respectively. No. of animals: HFD, 128 males, 102 females; Chow, 146 males, 137 females

**Fig. 2:**
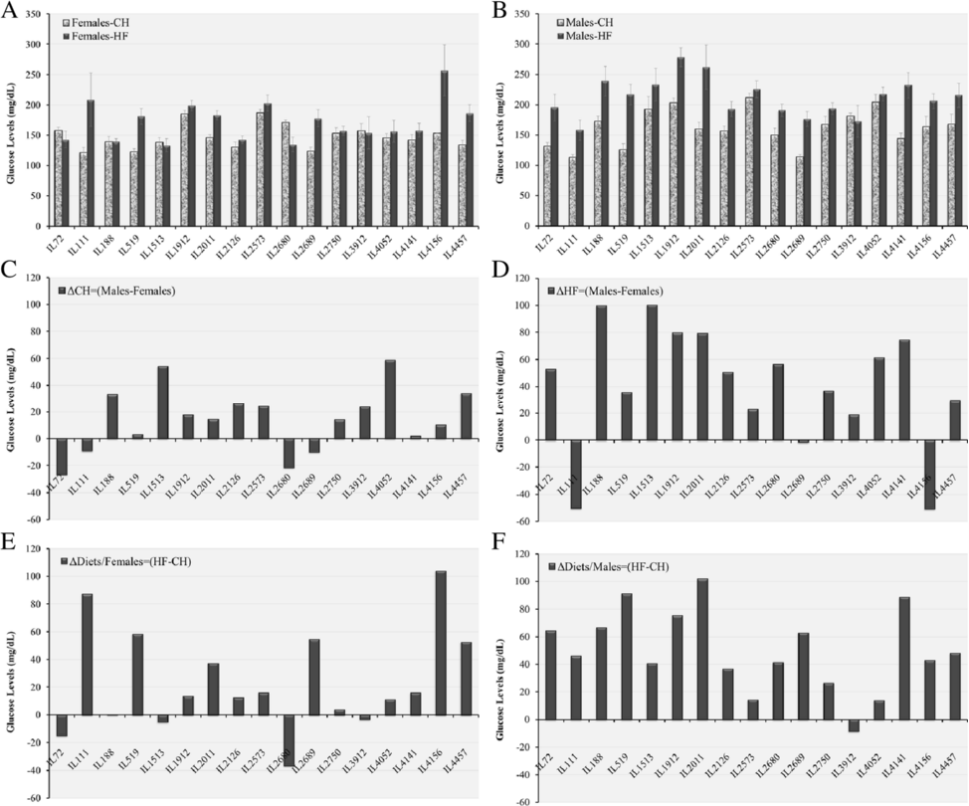
Fasting Glucose levels (mg/dL) of 17 CC lines separately for females and males, after 12 weeks on Chow diet (CH, 18 % fat) and on high fat diet (HFD, 42 % fat) measured at time 0 before IPGTT glucose injection. Bar graphs **a** and **b** show Fasting Glucose level means (±SE) for Chow and HFD of females and males, respectively, by lines. Bar graphs **c** and **d**, show diference between males and females for Chow (ΔCH = Males-Females) and HFD (ΔHF = Males-Females), respectively. Bar graphs **e** and **f** show differences between Chow and HFD for females (ΔDiets/Females = HF-CH) and males (ΔDiets/Males = HF-CH), respectively. No. of animals: HFD, 128 males, 102 females; Chow, 146 males, 137 females

**Fig. 3:**
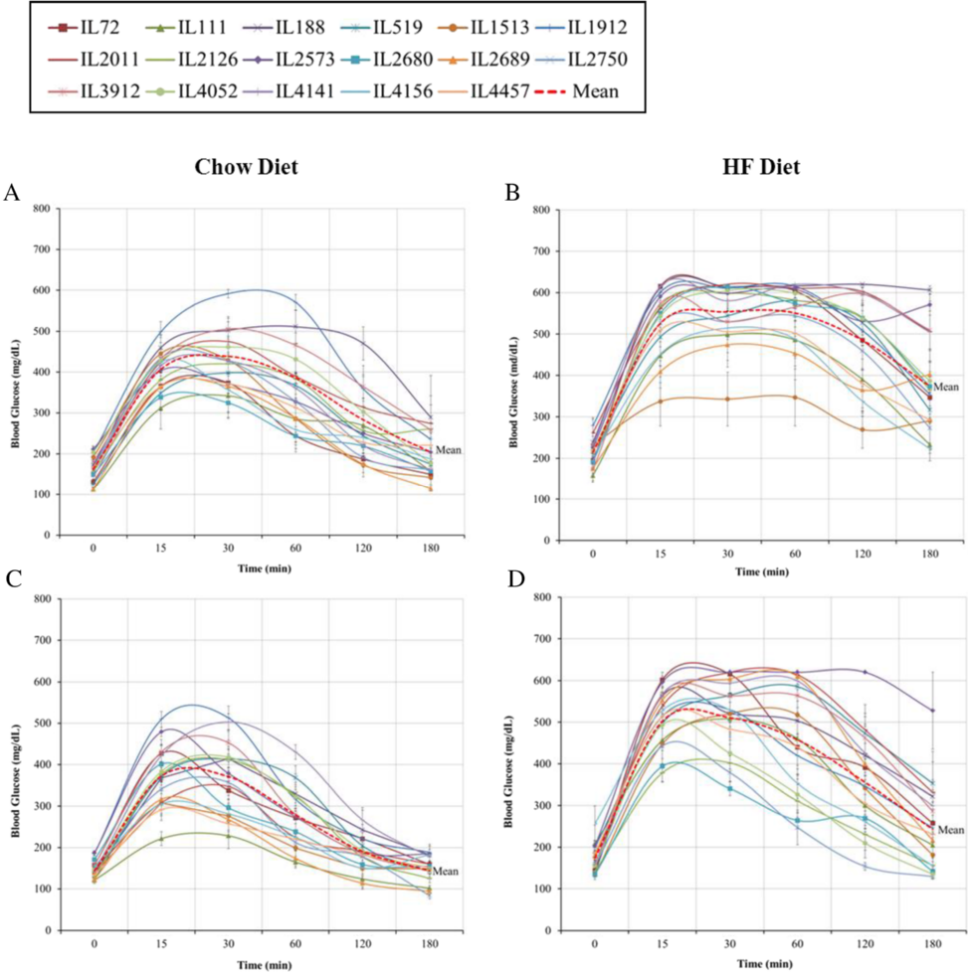
Kinetics of Intraperitoneal glucose tolerance test (IPGTT) showing mean blood glucose level (±SE) at different time points during 180 min test. Data are shown for 17 CC lines, separately for males and females, at age 20 weeks after 12 weeks on Chow diet (CH, 18 % fat) and on high fat diet (HFD, 42 % fat). Blood glucose levels were measured at time 0, 15, 30, 60, 120 and 180 min after glucose injection. The dark central line shows average glucose levels across lines. Chart **a**, Males-Chow; **b**, males-HFD; **c**, females-Chow; **d**, females-HFD. No. of animals: HFD, 128 males, 102 females; Chow, 146 males, 137 females

**Fig. 4:**
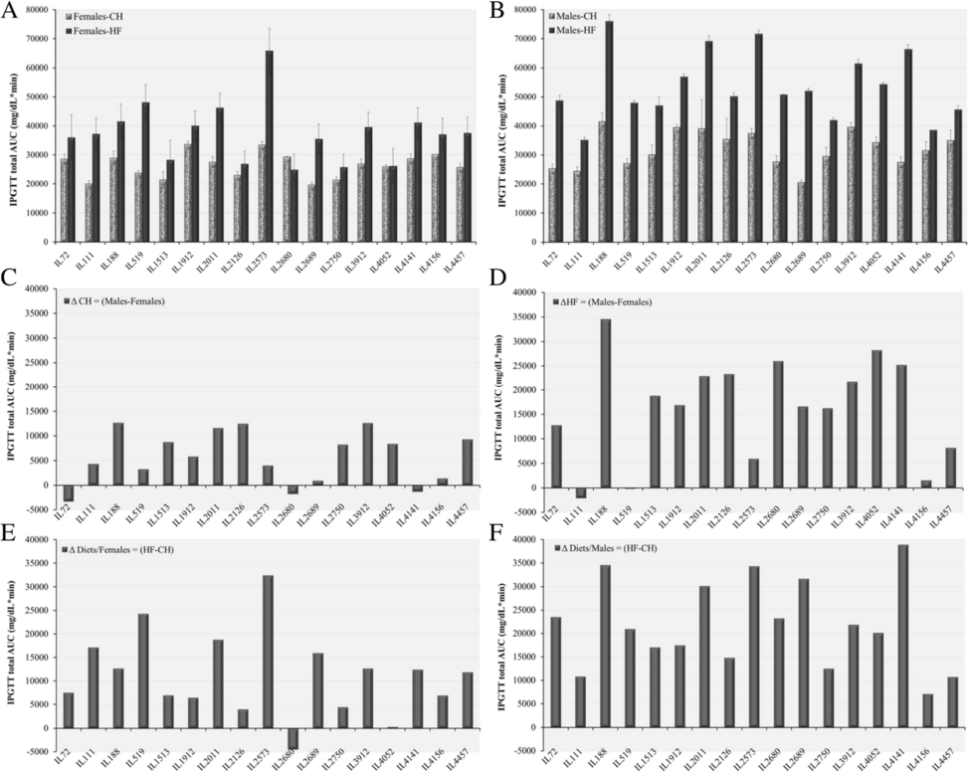
Blood glucose levels (mg/dL) during intraperitoneal glucose tolerance test (IPGTT) of 17 CC lines separately for females and males, after 12 weeks on Chow diet (18 % fat) and on high fat diet (HFD, 42 % fat) measured at time 0, 15, 30, 60, 120 and180 min after glucose injection. Bar graphs **a** and **b** show means (±SE) of total area under the curve (AUC) for Chow and HFD of females and males, respectively, by lines. Bar graphs **c** and **d**, show diference between males and females for Chow (ΔCH = Males-Females) and HFD (ΔHF = Males-Females), respectively. Bar graphs **e** and **f** show differences between Chow and HFD for females (ΔDiets/Females = HF-CH) and males (ΔDiets/Males = HF-CH), respectively. No. of animals: HFD, 128 males, 102 females; Chow, 146 males, 137 females

Table 3 presents global effect of diet and sex across all lines. To show effects of the individual lines, Figs. 1, 2, 3, and 4 present behavior of the individual lines by sex and diet with respect to BW, fasting blood glucose, IPGTT-AUC and kinetics of IPGTT. In all cases, the line x sex x diet values represent the mean values of a number of individuals of each sex in each line as given in Materials and Methods.

In Figs. 1, 2, 3, Bar Charts A and B represent trait values for males and females, respectively; Bar Charts C and D shows differences between males and females under Chow and HFD, respectively; and Bar Charts E and F show differences between Chow and HFD for females and males, respectively. Inspection of the bar charts of Figs. 1, 2 and 3 shows that within the general effects of sex and diet shown in Table 3, there was tremendous variation among lines for the actual trait values, and for the effects of sex and diet on trait values.

Figure 1 emphasizes the ability of females and males of some lines to maintain close to normal BW even under HFD. For females under HFD (Bar Chart E), 11 out of the 17 lines did not differ appreciably, or gained even less than females under Chow. Even for males six of the lines under HFD, did not differ appreciably or at all from their line-mates under Chow. For five of these lines, females also did not differ under Chow and HFD. Thus, these lines are apparently able to control BW gains even under HFD challenge.

Figure 2 presents Bar charts showing fasting blood glucose values for females and males of the individual CC lines after 12 weeks dietary challenge. Most female lines show minor response to HFD; with a few lines showing moderate to strong responses (see also Bar chart E). For males, all lines but one, show moderate to strong response to HFD (see also Bar chart F). Bar charts C, D show differences between sexes under Chow and HFD, respectively. Under Chow differences are small, but generally favor males. Under HFD, with two strong exceptions, males show clearly higher values than females.

Figure 3 presents graphs showing the kinetics of IPGTT for the individual CC lines separately for females and males, after 12 weeks dietary challenge. Blood glucose levels reached peak value at 20–30 min, and thereafter began to decline, either immediately or after a more or less extended plateau period. With some exceptions, initial 0 time levels were approached but not reached by the end of the test period (180) minutes. For both males and females there was a clear increase in peak glucose values under HFD, in addition to a major increase in post-peak plateau periods.

On average across lines under Chow diet, peak values for males were slightly greater than for females, but overall responses of males and females were within the same range. Under HFD peak, values of males and females increased by about the same amount averaged across lines. However, males averaged an appreciably longer post-peak plateau period than females. Remarkably, for the males, the variation among lines became smaller on HFD compared to Chow, while the females showed marked increase in between line variation on HFD. Males were uniformly affected by the HFD; females less so on average, but very variable, with some lines essentially unaffected, other lines reacting as strongly as males.

Figure 4 presents Bar charts showing total area under the curve (AUC) for Chow and HFD diets of females and males, respectively, by lines. With one exception, all lines responded to the HFD by an increase in AUC. The increase, for females was generally small, with one or two exceptions, while all male lines showed a large increase in AUC

Bar graphs C and D show difference between males and females under Chow diet (C) and HFD (D), respectively. Under Chow diet, males tend to show somewhat higher AUC than females, but the differences are small or negative. Converesly, under HFD most lines show a marked increase in males compared to females. Even here for a few lines the difference is small or even negative. Finally, bar graphs (E) and (F) show difference between HFD and Chow for females (E) and males (F). For females, differences are generally small, and in one case negative. For the males, all differences are postive and generally appreciable.

### Kinetics of BW and possibility of two-stage growth curves

**Fig. 5:**
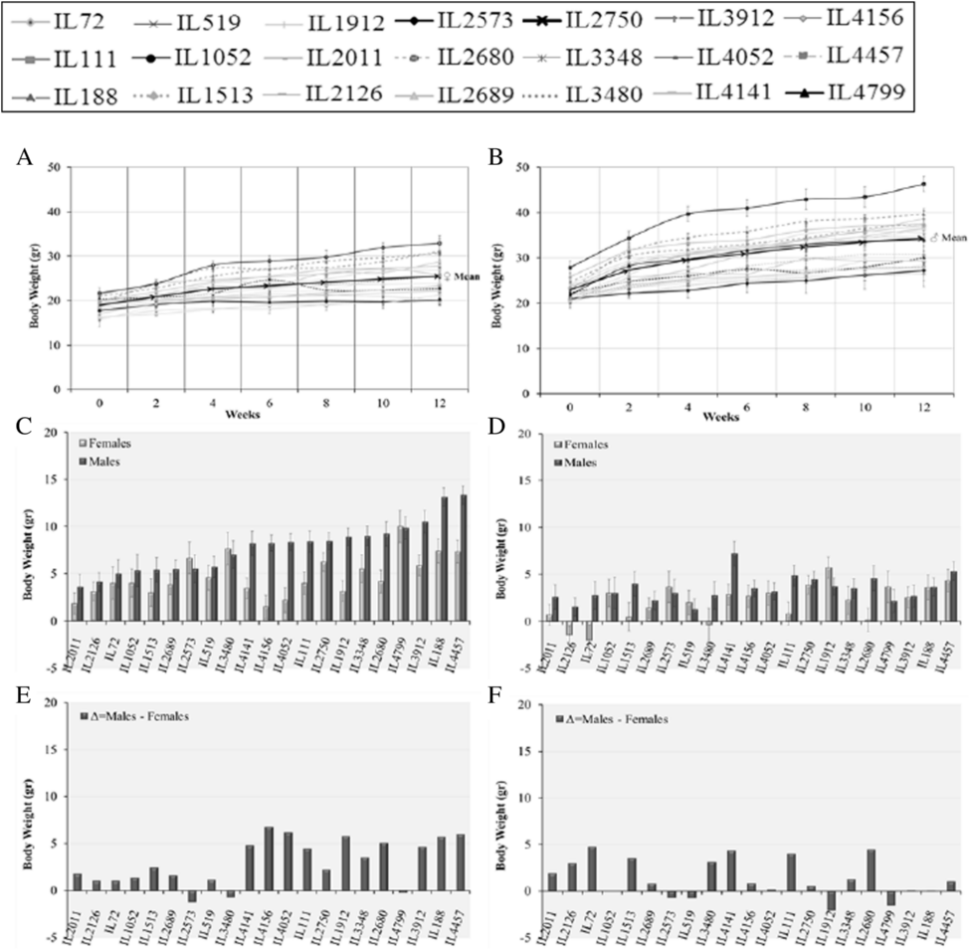
Kinetics of Body Weight (g) means (±SE) of females (**a**) and males (**b**) of 21 CC lines from 0 to 12 weeks on high fat diet (HFD, 42 % fat). **c** and **d**, bar graphs showing mean gains (±SE) on HFD of males and females weeks 0 to 6 and 7 to 12, respectively; lines ordered according to male gains from 0 to 6 weeks. **e** and **f**, bar graphs showing differences between males and females in weight gains from 0 to 6 and from 7 to 12 weeks on HFD, respectively. No. of animals: 149 males, 116 females

Figure 5 presents graphs and bar charts showing kinetics of BW of females and males of the individual CC lines from 8 to 20 weeks on HFD. The graphs show that both sexes gain weight for the first 6 weeks on HFD. For the second 6 weeks, while males of all lines continue to gain weight; some of the female lines do not gain weight or even lose weight.

Bar chart C shows the BW gains across the first 6 weeks on HFD, separately for males and females of the 21 CC lines. The CC lines are ordered according to male weight gain in this period and the gains of their female counterparts are shown alongside. Weight gains in the second 6 weeks are shown in Bar chart D. The difference in gain in the second 6-week period *(*Bar chart D*)* compared to the first 6-week period *(*Bar chart C*)* is dramatic; male weight gains were very small, and none were comparable to those in the first 6 weeks. Female weight gains ceased or were very slight; a number of lines even showing a small decrease in weight.

Bar chart E shows difference in body weight gain for first 6 weeks on HFD between males and females. Differences were small for the males and females of the low male-gain lines; much larger for the males and females of the moderate to high male-gain lines.

Bar charts D and F show corresponding values for the body weight gain on HFD second period, showing tendency for lines with high male gains in the first period to have higher gains in the second period for both sexes. The lines for which the females lost or gained little weight were concentrated in the lower half of the male lines in the first period. Bar chart F shows small or even negative differences between males and female gains for many lines, while for other lines differences were large. Interestingly, a few lines that show moderate increase in gains in the first period, show higher increase in the second period (e.g. IL2126, IL72, IL1513, IL3480). This phenomenon may reflect different metabolic mechanisms at different time points of development (see Discussion).

### One-way ANOVA, differences among lines

Figures 1–5 display extensive variation among CC lines for the measured phenotypes. Similar variation was also found for the other measured traits (data not shown). For significance of the variation, One-Way ANOVA for the complete set of traits was calculated, separately for each of the four sex x diet combinations. In all cases, differences among lines were significant at *P* < 0.05 to *P* < 0.001, and the extreme high and low value lines for any given trait differed significantly by Duncan’s multiple range test (see Additional file 1: Figure S1 and S2)

### Broad sense heritability and genetic coefficient of variation

Table 1 shows broad sense heritability (H^2^) and genetic coefficient of variation (CVg) of all tested traits; calculated separately by sexes and diets. CVg was not calculated for the “gain” traits, since the gain values varied from positive to negative. Consequently, the mean gain was small while genetic variation (*V*_*G*_) was large. Owing to this, the calculated values for CVg of the gain traits were often greater than 1.00 and were essentially meaningless. As noted above, initial trait values for both diets are the same. Heritability values were generally moderate, in the range 0.24 to 0.57. Heritabilities for a given sex x trait combination under Chow and HFD were generally quite similar. Heritabilities for females (mean 0.480) were greater than males (mean 0.344), under both diets. CVg values range was 0.08 to 0.27, but most values were in the range 0.09 to 0.20. CVg values for initial BW were always the lowest and those for AUC the highest, for any sex x diet combination.

Table 1 also shows H^2^n values calculated and adjusted for the number of animals in each sex x diet group. The H^2^n values are very high, indicating that the observed varation among the line trait-means is primarily due to genetic factors, and not to environmental factors. The differences in mean H^2^n values between sexes is mostly due to the different mean line numbers for males and females under the different diets, e. g., for females mean H^2^ is very similar under CH (0.49) and HFD (0.47); while H^2^n is appreciably less under HFD (0.83) than under CH (0.93), reflecting the larger number of females per line under CH (8.55) compared to HFD (5.52).

### Genetic correlations among the same trait in the two sexes

Table 2 shows phenotypic (rPnmf) and genetic (rGmf) correlations for the same traits in males and females. The cross sex genetic correlations for BW are high, indicating that all through the experimental period, 70 % to 80 % of the genetic factors controlling BW are common to males and females. Cross-sex genetic correlations for WC, fasting glucose and AUC are less, in the range 50 to 70 %, indicating influence of sex-specific factors. BMI shows very high cross-sex correlations for end BMI but very low correlation for initial BMI. Consequently, genetic correlation for BMI gain is also very low.

### Phenotypic correlations among different traits within sexes

Table 4 shows the phenotypic correlations among the various traits within sexes, separately for males and females. Considering females, there were moderate to high positive correlations among all body measurement traits, except initial BMI, which had low and even negative correlations with all other body and gain measurements. IPGTT had low correlations with most body traits, and slightly negative correlations with initial BW and initial BMI. However, there were high correlations with BW and BMI gain, although not with WC gain. Fasting glucose levels had moderate positive correlations with end BW and BW gain and high correlation (*r* = 0.597, *P* < 0.01) with Total AUC.

**Table 4:** Correlation matrix between line means of all traits on high-fat dietary challenge (HFD, 42 % fat) within sexes (females, above the diagonal; males, below the diagonal) ^a^. BW, Body Weight; BMI, Body Mass Index; WC, Waist circumference; Total AUC, total area under curve of the intraperitoneal glucose tolerance test; Initial, measurements at experiment start-point age of 8 weeks; End, end time-point of the experiment after 12 weeks high-fat dietary challenge; Gain, difference between end and initial time-point values

Trait	BW Initial	BMI Initial	WC Initial	BW End	BMI End	WC End	BW Gain	BMI Gain	WC Gain	Fasting Glucose	Total AUC
BW Initial	1	0.227	0.520*	0.636**	0.507*	0.550**	0.280	0.266	0.148	0.003	−0.171
BMI Initial	0.435*	1	0.010	−0.110	0.188	−0.153	−0.253	−0.557**	−0.165	−0.142	−0.350
WC Initial	0.728**	0.301	1	0.406	0.506*	0.417	0.240	0.421	−0.371	0.172	0.254
BW End	0.875**	0.473*	0.660**	1	0.778**	0.828**	0.919**	0.737**	0.522*	0.208	0.345
BMI End	0.778**	0.342	0.688**	0.827**	1	0.678**	0.709**	0.711**	0.290	0.070	0.274
WC End	0.735**	0.446*	0.591**	0.816**	0.715**	1	0.749**	0.684**	0.690**	0.094	0.379
BW Gain	0.710**	0.443*	0.551**	0.962**	0.765**	0.773**	1	0.781**	0.574**	0.257	0.517*
BMI Gain	0.594**	−0.155	0.568**	0.626**	0.875**	0.522*	0.576**	1	0.363	0.161	0.482*
WC Gain	0.355	0.322	−0.032	0.506*	0.360	0.788**	0.537*	0.213	1	−0.041	0.185
Fasting Glucose	−0.121	0.176	−0.315	−0.085	−0.310	0.020	−0.056	−0.416	0.265	1	0.597**
Total AUC	−0.347	0.101	−0.171	−0.106	−0.316	−0.026	0.042	−0.384	0.098	0.585**	1

^a^ No. of animals: 21 lines all HFD, 149 males, 116 females

*, *P* < 0.05; **, *P* < 0.01

Considering the males, there were moderate to high positive correlations among all of the body traits. Here too, initial BMI was lowly correlated with other traits, and negatively correlated with BMI gain. High correlations were found between initial BW and the other body measurements. Indeed, correlations among the body traits were generally higher for the males than for the females, especially for correlations of initial and end BW with other traits. Correlations between IPGTT and body traits were preponderantly low negative, with some low positive. IPGTT correlated negatively with BMI gain, and low positively with BW and WC gain; similarly for Fasting glucose levels.

Summarizing main inferences of Table 4, initial BW explained most variation in body traits in males, while in females it was end BW. Fasting glucose levels and total AUC were highly positive correlated in both sexes. BW gain and BMI gain were predictive of AUC in females, but not in males.

Overall, the extremely susceptible CC lines towards development of both obesity and glucose tolerance in response to HF dietary challenge are IL-4457, IL-188, IL-1513, IL-2573, IL-4141 and IL-2750 of both sexes. Additional CC lines with similar susceptibility but sex-dependent are, females of IL-519, IL-3912 and males of IL-1912, IL-2011, IL-2680, and IL-4052. The only extremely resistible CC lines towards, both development of obesity and impaired glucose tolerance in response to HF diet were only females of IL-2680 and IL-4052 (moderate). On other hand, CC lines that exhibit extreme resistibility towards obesity with extreme/moderate susceptibility towards impaired glucose tolerance are, IL-72, IL-111, IL-2126 and IL-2689 of both sexes, only males of IL-519 and IL3912, and only females of IL-1912, IL-2011 and IL-4156.

## Discussion

Metabolic syndrome (MTS) is a collection of risk factors, including central obesity, reduced HDL;along with high blood pressure, triglycerides, and fasting blood glucose levels, that increase likelihood of T2D [51, 52]. The current model for the causative pathway, leads from BW gain in response to high fat diet (HFD) to central obesity, and from there to insulin resistance and high fasting blood-glucose levels [44, 53]. The latter are thought to have inflammatory effects on many body systems, leading to T2D and metabolic syndrome complications, expressed as insulin resistance with inflammation-impaired pancreatic beta-cell function reflected as impaired Glucose tolerance during an intraperitoneal glucose tolerance test (IPGTT) [54, 55].

The major objective of the present study was to assess the suitability of the novel and genetically highly diverse mouse CC genetic reference population [39, 61, 62], as a model system for exploring genetics of MTS and T2D. For this, we compared responses to HFD and standard Chow diets with respect to development of these conditions in cohorts of the CC lines. We monitored body weight gain, abdominal obesity as measured by body mass index (BMI) and body waist circumference (WC) due to their central role in etiology of MTS; and fasting glucose levels and IPGTT test for glucose tolerance measured by total area under the curve (AUC). We asked whether HFD induces changes typical of MTS and T2D; whether there were sex differences in response to HFD as found in humans; and whether there was abundant genetic variation to provide good chances for successful genomic analysis to the level of the individual gene.

### Dietary effects on development of impaired fasting glucose and IPGTT in the CC mice

Mice reared on the HFD showed clear development of physical and biochemical biomarkers for MTS, which agreed with a previously published study [63]. However, in our study, development of the physical markers was much more pronounced for males than for females. As shown in Table 3, under HFD, males presented gains in BW, BMI and WC that were, respectively 2.00-, 3.15-, and 1.70-fold greater than for males under Chow diet. The corresponding values for females were 1.53, 1.27-, and 0.83-fold thus aside from BW gain, HFD had little effect on physical markers in females. This sex difference may be a reflection of the greater BW gains of the males under HFD (two-fold greater than under Chow) as compared to females (only 1.53-fold greater than under Chow), which might be sufficient to tip males over the edge to obesity while keeping females lean. In contrast, the strong effect of HFD on fasting glucose levels for females (relative to Chow) was proportionately the same as for males. Thus, HFD was very effective in generating MTS-like condition in both sexes as judged by insulin resistance. Similarly, an accepted diagnostic criterion for T2D is impaired glucose clearance after high glucose load challenge by intraperitoneal glucose injection. Figure 3 shows that for the most part, on Chow diet both sexes succeed in returning glucose levels close to normal by 180 min following IPGTT challenge; while the mice on HFD mice failed to do so. Total AUC from 0 to 180 min as a quantitative measure for glucose tolerance reveals that on average across all lines for both sexes, the HFD mice showed significantly reduced glucose tolerance, as shown by greatly increased AUC relative to standard Chow diet mice. Thus, HFD clearly induces a T2D-like condition in these mice, and was only a bit less effective in females as in males.

### Phenotypic and genetic correlations among traits

Examining the full matrix of phenotypic correlations within sexes for the different traits in response to HFD presents an intriguing picture (Table 4). For both sexes, there is a strong positive correlation between BW gain and obesity measures (BMI and WC). In females but not males, this continues with positive association of BW gain, BMI gain (although not WC gain) with fasting blood glucose levels and AUC. This is consistent with the suggestion that increase in body weight is a key abnormality predisposing to insulin resistance [64, 65]. Remarkably, in males, BMI was negatively correlated (although not significantly) with Glucose tolerance traits. We speculate that this may to be due to threshold effects of BW gain on development of insulin resistance. To wit, that below a certain threshold, there is a monotonic relation between obesity (as measured by BMI) or BW gain and insulin resistance; but above this level, BW gain has exerted its maximum effect, and there is no further relation between degrees of BW gain and degree of insulin resistance. We propose that for females, development of obesity for most CC lines under our experimental conditions is below the threshold, exposing the positive correlation between obesity and insulin resistance; while for males, development of obesity for most lines is above the threshold.

Genetic correlations between the physical markers across the two sexes were high for BW and WC; somewhat erratic for BMI (Table 2). Thus, for the most part the same genetic factors appear to govern the effect of HFD on BW and obesity by WC and BMI in the two sexes, although the absolute impact of HFD on obesity differs between sexes, as described above. The situation is different for fasting glucose and AUC, with cross-sex genetic correlations equal 0.497 and 0.670, respectively. For these traits, there appears to be a greater presence of sex-specific genetic factors that modify the response to HFD.

### Genetic variation in dietary effects among the CC lines

The dietary and sex effects discussed above represent overall average effects of diet and sex, with wide variation among the lines, as emphasized in the graphs of BW kinetics of the CC lines (Fig. 5) and Glucose clearance kinetics during IPGTT (Fig. 3). More detailed bar charts presentations (Figs. 1, 2 and 4), show wide variation among lines in end BW, fasting glucose and total AUC. The variation is expressed in absolute values of males and females of the individual lines according to diet; in differences between males and females by diets; and in llldifferences in dietary effects in males and in females.

Under Chow diet, for 6 of 17 lines, both sexes were able to return blood glucose levels to initial fasting levels by the end of the IPGTT test at 180 min. The same was found for females but not males of 3 additional lines. Under HFD, for 6 of the lines, females were able to return to initial levels at 180 min, but for none of the lines were the males able to do so. Even of the 6 lines for which females returned to starting levels under HFD, five showed greater fasting glucose levels under HFD under Chow diet, for females. Thus, there was one line where females maintained normal fasting levels and returned to initial levels after challenge under HFD. This line also had males that returned to initial levels at 180 min under Chow, but not under HFD. Thus, this was a very resistant line, but still susceptible to HFD induced impaired glucose metabolism to some extent.

Broad sense heritability coefficients (H^2^) based on individual mice are in the moderate range, indicating that at the level of the individual mouse, the target traits are affected more or less equally by genetic and environmental factors and their interaction. At the level of the line, however, heritability coefficients (H^2^n) are very high, indicating that the observed variation among line-means described above, is primarily genetic in origin. The genetic coefficient of variation (CV_G_) ranges from 0.07 to 0.27, but most values are in the range 0.12 to 0.17. This is about twice the value found for most quantitative traits in wild or domesticated animal populations. Thus, in this case also, the CC lines appear to have incorporated a massive amount of genetic diversity. In all likelihood, this is due to the inclusion of the three wild-derived strains and subspecies among the founder lines of the CC population. For all diet x sex combinations, CV_G_ was least for initial BW, and greatest for AUC. Initial BW (i.e., BW under standard diet) is under strong stabilizing selection in the wild, and would not be expected to show a great deal of genetic variation, even in wild accessions. In contrast, AUC is a trait that would not be under any degree of selection in the wild, and hence can be expected to show a great deal of variation when exposed to challenge. These effects parallel to the well-known observation that naïve human populations exposed to the Western diet tend to develop extreme obesity and MTS health complications. It would be of interest to explore whether there is also great variation in degree of obesity in these populations.

### Categorical or quantitative?

The susceptibility of different mouse strains to diet-induced obesity and insulin resistance will be partly related to genetic difference in variables linked to energy balance and glucose homeostasis [66–68]. In addition, the response of different mouse strains will be highly dependent on feeding duration, diet composition and housing conditions. Variations in these experimental parameters may explain why previous studies have characterized commercial inbred lines as either prone to obesity and insulin resistance [28, 66], or relatively resistant to the effects accompanying an HFD [66, 69]. The strength of our study is that all CC lines were studied under the same experimental conditions and both sexes were analyzed, as well. Comparing the various lines, it is clear that the variation in our population of lines is quantitative rather than categorical. That is, the lines differ in a quantitative way along a spectrum of values for the various traits measures. The differences among lines with respect to the measured traits do not behave in an “all or none” manner

### Two-stage regulation of body weight gain

**Fig. 6:**
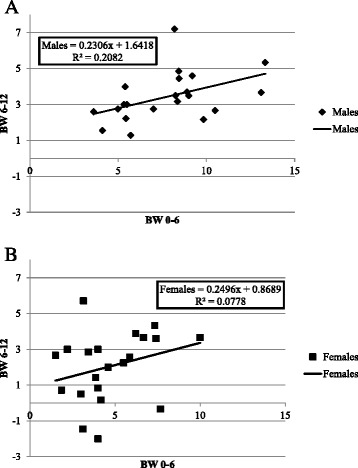
Scattergrams showing regression of Body Weight (BW) gain in weeks 7 – 12 on Body Weight (BW) gain in weeks 0–6 on High-Fat diet (HFD, 42 % fat) by line means for 21 lines. Chart **a**, males; **b**, females. No of animals, 149 males, 116 females

As noted, the major body weight changes were observed during the first 6 weeks on HFD in both sexes, with higher gains in males as shown Fig. 5. These changes were less in the remaining 6 weeks of the experiment. After 6 weeks on HFD females appear to be able to defend against further increase in BW, while males continue to increase in BW over the entire experimental period. Figure 6a (males) and b (females) present the regression of BW gain in weeks 7–12 on BW gain in weeks 0–6 on HFD challenge for both sexes, showing a moderate correlation in males, but essentially zero correlation for females. Thus, main body weight increases on HFD challenge occurs during weeks 0–6, while the total result of body weight accumulation can be set by the body weight dynamics during the 2nd period. We speculate that there are 3 main options during 2nd period: 1) High body weight defense by body weight decline shown by some female lines; 2) Moderate body weight defense by inhibiting/restraining body weight increase shown some females and males lines and 3) Continuous body weight increase since challenge start point. In females, the choice of options appears to be independent of gains in the first 6 weeks, while in males there is a moderate correlation with first 6-week gains but considerable leeway for individual variation. These sex differences might be due to the different hormone expression variations in both sexes, as suggested in human studies [17, 70, 71]

## Conclusions

Under Chow diet, both sexes of all lines did not show impaired IPGTT tests. There was very wide statistically significant variation among the individual lines in their response to HFD (Figs. 1–5). Broad sense heritability coefficients (H^2^) at the level of the individual mouse are in the moderate range (Table 1), indicating that target traits are affected more or less equally by genetic and environmental factors and their interaction. At the level of line, however, heritability coefficients (H^2^n) are very high, indicating that observed variation among line-means is primarily genetic in origin. Genetic coefficient of variation (CV_G_) of CC population is about twice the value than in quantitative traits of wild or domesticated animal populations, probably due to the inclusion of the wild accessions in the formation of the CC lines. Two-stage control of weight gains were shown under HFD, the first 6 weeks, where both sexes of all lines gained weight, and second 6 weeks, where most male lines continued to gain weight, while the females varied in line behavior - some lost weight, some maintained weight, and only a few continued to gain weight. Females were much more able to control BW gains under HFD. Our results demonstrates the important role of sex differences in the physiology of MTS and T2D, that must be considered in advanced studies [72, 73].

Our results confirm that host susceptibility to high fat diet-induced T2D is a complex trait controlled by multiple genetic factors and that the CC population will be a powerful and promising tool for dissecting this trait, as shown in previous studies of other traits [40, 42, 53, 74, 75].

## Additional file

Additional file 1: Figure S1. Duncan’s analysis for body weight gain means (±SE) 0 to 12 weeks of males on high fat diet (HFD, 42% fat). No. of animals: 149 males). Figure S2 Duncan’s analysis for total AUC means (±SE) of males on high fat diet (HFD, 42% fat). No. of animals: 149 males). (PDF 331 kb)

## References

[1] National Diabetes Information Clearinghouse. National Diabetes Statistics. NIH Publication. http://www.cdc.gov/diabetes/pubs/statsreport14/national-diabetes-report-web.pdf. (2014). Accessed 26 Apr 2015.

[2] Narayan KM, Boyle JP, Thompson TJ, Sorensen SW, Williamson DF (2003). Lifetime risk for diabetes mellitus in the United States. JAMA.

[3] Zimmet P, Alberti KG, Shaw J (2001). Global and societal implications of the diabetes epidemic. Nature.

[4] Florez JC, Hirschhorn J, Altshuler D (2003). The inherited basis of diabetes mellitus: implications for the genetic analysis of complex traits. Annu Rev Genomics Hum Genet.

[5] Permutt MA, Wasson J, Cox N (2005). Genetic epidemiology of diabetes. J Clin Invest.

[6] van Tilburg J, van Haeften TW, Pearson P, Wijmenga C (2001). Defining the genetic contribution of type 2 diabetes mellitus. J Med Genet.

[7] Das SK, Elbein SC (2006). The genetic basis of type 2 diabetes. Cellscience.

[8] Duggirala R, Blangero J, Almasy L, Dyer TD, Williams KL, Leach RJ (1999). Linkage of type 2 diabetes mellitus and of age at onset to a genetic location on chromosome 10q in Mexican Americans. Am J Hum Genet.

[9] Sladek R, Rocheleau G, Rung J, Dina C, Shen L, Serre D (2007). A genome-wide association study identifies novel risk loci for type 2 diabetes. Nature.

[10] Moore AF, Jablonski KA, McAteer JB, Saxena R, Pollin TI, Franks PW (2008). Diabetes Prevention Program Research Group. Extension of type 2 diabetes genome-wide association scan results in the diabetes prevention program. Diabetes.

[11] Bowden DW, Rudock M, Ziegler J, Lehtinen AB, Xu J, Wagenknecht LE (2006). Coincident linkage of type 2 diabetes, metabolic syndrome, and measures of cardiovascular disease in a genome scan of the diabetes heart study. Diabetes.

[12] Legato MJ, Gelzer A, Goland R, Ebner SA, Rajan S, Villagra V (2006). Sex-specific care of the patient with diabetes: review and recommendations. Gend Med.

[13] Institute of Medicine (US) Forum on Neuroscience and Nervous System Disorders. Sex Differences and Implications for Translational Neuroscience Research: Workshop Summary. Washington (DC): National Academies Press (US). The National Academies. http://www.ncbi.nlm.nih.gov/books/NBK53385/ (2011). Accessed 20 April 2015. 21452459

[14] Hilawe E, Yatsuya H, Kawaguchi L, Aoyama A (2013). Differences by sex in the prevalence of diabetes mellitus, impaired fasting glycaemia and impaired glucose tolerance in sub-Saharan Africa: a systematic review and meta-analysis. Bull World Health Organ.

[15] Church DM, Goodstadt L, Hillier LW (2009). The Mouse Genome Sequencing Consortium" Lineage-Specific Biology Revealed by a Finished Genome Assembly of the Mouse. PLoS Biol.

[16] NIH-National Human Genome Research Institute. The Mouse Genome And the Measure of Man. NIH News Advisory. http://www.nih.gov/news/pr/dec2002/nhgri-04.htm(2002). Accessed 20 April 2015.

[17] Gunter C, Dhand R (2002). The mouse genome: Human biology by proxy. Nature.

[18] Lander ES, Schork NJ (1994). Genetic dissection of complex traits. Science.

[19] Paterson AH (1995). Molecular dissection of quantitative traits: progress and prospects. Genome Res.

[20] Vidal SM, Malo D, Vogan K, Skamene E, Gros P (1993). Natural resistance to infection with intracellular parasites: isolation of candidate for Bcg. Cell.

[21] Blackwell MJ, Barton CH, White KJ, Searle S, Baker AM, Williams H (1995). Genomic organization and sequence of human NRAMP gene: identification and mapping of a promoter region polymorphism. Mol Med.

[22] Flint J, Valdar W, Shifman S, Mott R (2005). Strategies for mapping and cloning quantitative trait genes in rodents. Nat Rev Genet.

[23] Machleder D, Ivandic B, Welch C, Castellani L, Reue K, Lusis AJ (1997). Complex genetic control of HDL levels in mice in response to an atherogenic diet. Coordinate regulation of HDL levels and bile acid metabolism. J Clin Invest.

[24] Korstanje R, Li R, Howard T, Kelmenson P, Marshall J, Paigen B (2004). Influence of sex and diet on quantitative trait loci for HDL cholesterol levels in an SM/J by NZB/BlNJintercross population. J Lipid Res.

[25] Ishimori N, Li R, Kelmenson PM, Korstanje R, Walsh KA, Churchill GA (2004). Quantitative trait loci analysis for plasma HDL-cholesterol concentrations and atherosclerosis susceptibility between inbred mouse strains C57BL/6 J and 129S1/SvImJ. Arterioscler Thromb Vasc Biol.

[26] Wang X, Paigen B (2002). Quantitative trait loci and candidate genes regulating HDL cholesterol: a murine chromosome map. Arterioscler Thromb Vasc Biol.

[27] Wang X, Ishimori N, Korstanje R, Rollins J, Paigen B (2005). Identifying novel genes for atherosclerosis through mouse-human comparative genetics. Am J Hum Genet.

[28] Andrikopoulos S, Massa CM, Aston-Mourney K, Funkat A, Fam BC, Hull RL (2005). Differential effect of inbred mouse strain (C57BL/6, DBA/2, 129T2) on insulin secretory function in response to a high fat diet. J Endocrinol.

[29] Berglund ED, Li CY, Poffenberger G, Ayala JE, Fueger PT, Willis SE (2008). Glucose metabolism in vivo in four commonly used inbred mouse strains. Diabetes.

[30] Boudina S, Sena S, Sloan C, Tebbi A, Han YH, O'Neill BT (2012). Early mitochondrial adaptations in skeletal muscle to diet-induced obesity are strain dependent and determine oxidative stress and energy expenditure but not insulin sensitivity. Endocrinology.

[31] Goren HJ, Kulkarni RN, Kahn CR (2004). Glucose homeostasis and tissue transcript content of insulin signaling intermediates in four inbred strains of mice: C57BL/6, C57BLKS/6, DBA/2, and 129X1. Endocrinology.

[32] Paigen B (1995). Genetics of responsiveness to high-fat and high cholesterol diets in the mouse. Am J ClinNutr.

[33] Leiter EH (2009). Selecting the “Right” mouse model for metabolic syndrome and type 2 diabetes. T2 Diabetes. Methods Mol Biol.

[34] Lin C, Theodorides ML, McDaniel AH, Tordoff MG, Zhang Q, Li X (2013). QTL analysis of dietary obesity in C57BL/6byj X 129P3/J F2 mice: diet- and sex-dependent effects. PLoS One.

[35] Keane TM, Goodstadt L, Danecek P, White MA, Wong K, Yalcin B (2011). Mouse genomic variation and its effect on phenotypes and gene regulation. Nature.

[36] Roberts A, Pardo-Manuel de Villena F, Wang W, McMillan L, Threadgill DW (2007). The polymorphism architecture of mouse genetic resources elucidated using genome-wide resequencing data: implications for QTL discovery and systems genetics. Mamm Genome.

[37] Churchill G, Airey D, Allayee H, Angel JM, Attie AD, Beatty J (2004). Complex Trait Consortium. The Collaborative Cross, a community resource for the genetic analysis of complex traits. Nat Genet.

[38] Yalcin B, Flint J, Mott R (2005). Using progenitor strain information to identify quantitative trait nucleotides in outbred mice. Genetics.

[39] Iraqi F, Churchill G, Mott R (2008). The Collaborative Cross, developing a resource for mammalian systems genetics: a status report of the Wellcome Trust cohort. Mamm Genome.

[40] Durrant C, Tayem H, Yalcin B, Cleak J, Goodstadt L, de Villena FP (2011). Collaborative Cross mice and their power to map host susceptibility to Aspergillus fumigatus infection. Genome Res.

[41] Aylor DL, Valdar W, Foulds-Mathes W, Buus RJ, Verdugo RA, Baric RS (2011). Genetic analysis of complex traits in the emerging Collaborative Cross. Genome Res.

[42] Philip VM, Sokoloff G, Ackert-Bicknell CL, Striz M, Branstetter L, Beckmann MA (2011). Genetic analysis in the Collaborative Cross breeding population. Genome Res.

[43] Chan JM, Rimm EB, Colditz GA, Stampfer MJ, Willett WC (1994). Obesity, fat distribution, and weight gain as risk factors for clinical diabetes in men. Diabetes Care.

[44] Carey VJ, Walters EE, Colditz GA, Solomon CG, Willett WC, Rosner BA (1997). Body fat distribution and risk of non-insulin-dependent diabetes mellitus in women. The Nurses’ Health Study. Am J Epidemiol.

[45] Wang Y, Rimm EB, Stampfer MJ, Willett WC, Hu FB (2005). Comparison of abdominal adiposity and overall obesity in predicting risk of type 2 diabetes among men. Am J Clin Nutr.

[46] American Diabetes Association. 2. Classification and Diagnosis of Diabetes. Diabetes Care 2015;38(Suppl. 1):S8–S16 | DOI: 10.2337/dc15-S005. 10.2337/dc15-S005 25537714

[47] World health Organization (2006). Definition and diagnosis of diabetes mellitus and intermediate hyperglycaemia, Report of a WHO/IDF consultation.

[48] e-biomethods. Intraperitoneal glucose tolerance test (IPGTT). http://www.ebiomethods.com. Accessed 20 April 2015

[49] Montgomery MK, Hallahan NL, Brown SH, Liu M, Mitchell TW, Cooney GJ (2013). Mouse strain-dependent variation in _obesity_ and glucose homeostasis in response to high-fat-feeding. Diabetologia.

[50] Iraqi FA, Athamni H, Dorman A, Salymah Y, Tomlinson I, Nashif A (2014). Heritability and coefficient of genetic variation analyses of phenotypic traits provide strong basis for high-resolution QTL mapping in the Collaborative Cross mouse genetic reference population. Mamm Genome.

[51] Garcia-Gonzalez F, Simmons LW, Tomkins JL, Kotiaho JS, Evans JP (2012). Comparing evolvabilities: common errors surrounding the use and calculation of coefficients of additive genetic variation. Evolution.

[52] Houle D (1992). Evolvability and variability of quantitative traits. Genetics.

[53] Vered K, Durrant C, Mott R, Iraqi FA (2014). Susceptibility to *klebsiella pneumonaie* infection in collaborative cross mice is a complex trait controlled by at least three loci acting at different time points. BMC Genomics.

[54] Robertson A (1955). Prediction equations in quantitative genetics. Biometrics.

[55] Falconer DS, Mackay TFC (1996). Quantitative Genetics 4th edition. Introduction to Quantitative Genetics.

[56] Olufadi R, Byrne CD (2008). Clinical and laboratory diagnosis of the metabolic syndrome. J Clin Pathol.

[57] Hanley AJ, Karter AJ, Williams K, Festa A, D'Agostino RB, Wagenknecht LE (2005). Prediction of type 2 diabetes mellitus with alternative definitions of the metabolic syndrome: the Insulin Resistance Atherosclerosis Study. Circulation.

[58] Gustafson B, Hammarstedt A, Andersson CX, Smith U (2007). Inflamed adipose tissue: a culprit underlying the metabolic syndrome and atherosclerosis. Arterioscler Thromb Vasc Biol.

[59] Gami AS, Witt BJ, Howard DE, Erwin PJ, Gami LA, Somers VK (2007). Metabolic syndrome and risk of incident cardiovascular events and death: a systematic review and meta-analysis of longitudinal studies. J Am Coll Cardiol.

[60] Stehouwer CD, Henry RM, Ferreira I (2008). Arterial stiffness in diabetes and the metabolic syndrome: a pathway to cardiovascular disease. Diabetologia.

[61] Chesler EJ, Miller DR, Branstetter LR (2008). The Collaborative Cross at Oak Ridge National Laboratory: developing a powerful resource for systems genetics. Mamm Genome.

[62] Morahan G, Balmer L, Monley D (2008). Establishment of “The Gene Mine”: a resource for rapid identification of complex trait genes. Mamm Genome.

[63] Joost HG, Schürmann A (2014). The genetic basis of obesity-associated type 2 diabetes (diabesity) in polygenic mouse models. Mamm Genome.

[64] Cho YR, Kim HJ, Park SY, Ko HJ, Hong EG, Higashimori T (2007). Hyperglycemia, maturity-onset obesity, and insulin resistance in NONcNZO10/LtJ males, a new mouse model of type 2 diabetes. Am J Physiol Endocrinol Metab.

[65] Kim JH, Stewart TP, Zhang W, Kim HY, Nishina PM, Naggert JK (2005). Type 2 diabetes mouse model TallyHo carries an obesity gene on chromosome 6 that exaggerates dietary obesity. Physiol Genomics.

[66] Fearnside JF, Dumas ME, Rothwell AR, Wilder SP, Cloarec O, Toye A (2008). Phylometabonomic patterns of adaptation to high fat diet feeding in inbred mice. PLoS One.

[67] Carter CP, Howles PN, Hui DY (1997). Genetic variation in cholesterol absorption efficiency among inbred strains of mice. J Nutr.

[68] Mouse Phenome Database at the Jackson Laboratory. The Jackson Laboratory, Maine USA. https://www.phenome.jax.org. Accessed 26 April 2015.

[69] Almind K, Kahn CR (2004). Genetic determinants of energy expenditure and insulin resistance in diet-induced obesity in mice. Diabetes.

[70] Rathmann W, Strassburger K, Giani G, Döring A, Meisinger C (2008). Differences in height explain sex differences in the response to the oral glucose tolerance test. Diabet Med.

[71] Wallace IR, McKinley MC, Bell PM, Hunter SJ (2013). Sex hormone binding globulin and insulin resistance. Clin Endocrinol (Oxf).

[72] Clayton JA, Collins FS (2014). Policy: NIH to balance sex in cell and animal studies. Nature.

[73] IOM (Institute of Medicine) (2001). Exploring the biological contributions to human health: Does sex matter?.

[74] Bottomly D, Ferris MT, Aicher LD, Rosenzweig E, Whitmore A, Aylor DL (2012). Expression quantitative trait loci for extreme host response to influenza A in pre-collaborative cross mice. G3.

[75] Rasmussen AL, Okumura A, Ferris MT, Green R, Feldmann F, Kelly SM (2014). Host genetic diversity enables Ebola hemorrhagic fever pathogenesis and resistance. Science.

