# Supplementary material for: High-fat-diet induced development of increased fasting glucose levels and impaired response to intraperitoneal glucose challenge in the collaborative cross mouse genetic reference population
Source: BMC Genet. 2016 Jan 5;17:10. doi: 10.1186/s12863-015-0321-x (PMC4700737; doi:10.1186/s12863-015-0321-x)
Supplement: Additional file 1: Figure S1. — Duncan’s analysis for body weight gain means (±SE) 0 to 12 weeks of males on high fat diet (HFD, 42% fat). No. of animals: 149 males). Figure S2 Duncan’s analysis for total AUC means (±SE) of males on high fat diet (HFD, 42% fat). No. of animals: 149 males). (PDF 331 kb) [file 12863_2015_321_MOESM1_ESM.pdf]

Supplementary:

Figure 1. Duncan's analysis for body weight gain means (±SE) 0 to 12 weeks of males on high fat diet (HFD, 42% fat). No. of animals: 149 males).

Figure 2. Duncan's analysis for total AUC means (±SE) of males on high fat diet (HFD, 42% fat). No. of animals: 149 males).

Supplementary 1 presents an illustrative Bar chart for male weight-gain of the 21 CC lines after 12 weeks on HFD. On Duncan's multiple range test, there were 7 homogeneous groups, with much overlap among the groups. However close examination of the figure shows that lines IL2011, IL2076, and IL72 on the low side, do not overlap with, and hence differ significantly from lines IL3012, IL188, and IL4457 on the high side. Supplementary 2 shows the corresponding bar chart for male IPGTT AUC. In this case, lines IL111, IL3480, IL4256 and IL2750 on the low side differ significantly from lines IL1052, IL4141, IL2011, IL2573, IL4799 and IL 188 on the high side. Roughly similar results were obtained with the other trait x sex x diet combinations (data not shown).

Figure 1:

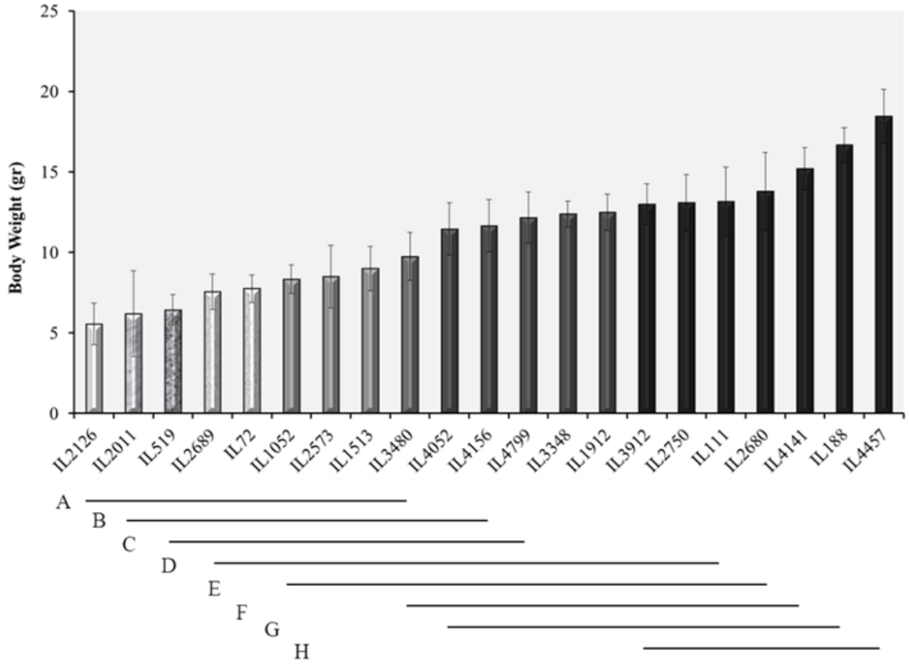

Figure 2:

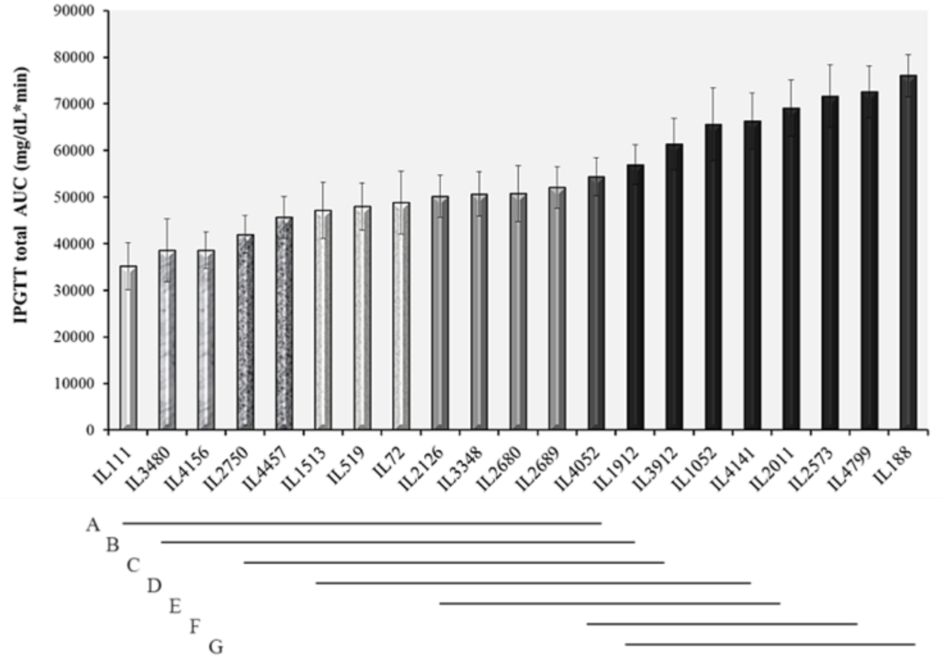
IPGTT total AUC (mg/dL*min)
90000
80000
70000
60000
50000
40000
30000
20000
10000
0
IL111
IL3480
IL4156
IL2750
IL4457
IL1513
IL519
IL72
IL2126
IL3348
IL2680
IL2689
IL4052
IL1912
IL3912
IL1052
IL4141
IL2011
IL2573
IL4799
IL188
A
B
C
D
E
F
G
